# Mapping behavioural mechanisms linking childhood maltreatment, cognition, impulsivity, and suicidality in bipolar disorder: A network approach

**DOI:** 10.1038/s41398-026-04177-1

**Published:** 2026-06-20

**Authors:** Natalia E. Fares-Otero, Astrid E. Iversen, Alfonso Gutiérrez-Zotes, Jeff Zarp, Esther Jiménez, Jose Sánchez-Moreno, Edith Pomarol-Clotet, Eduard Vieta, Hanne L. Kjærstad, Elisabet Vilella, Kamilla W. Miskowiak

**Affiliations:** 1 Department of Psychiatry and Psychotherapy, Technical University of Munich, TUM School of Medicine and Health, Klinikum rechts der Isar, Munich, Germany; 2 Department of Psychiatry and Psychology, Bipolar and Depressive Disorders Unit, Hospital Clínic, Institute of Neurosciences, University of Barcelona (UBNeuro), Barcelona, Spain; 3 Fundació de Recerca Clínic Barcelona, Institut d’Investigacions Biomèdiques August Pi i Sunyer (FRCB-IDIBAPS), Barcelona, Spain; 4 Faculty of Medicine and Health Sciences, University of Barcelona, Barcelona, Spain; 5 Neurocognition and Emotion Across Disorders of the Brain (NEAD) Centre, Psychiatric Centre Copenhagen, Frederiksberg Hospital, Copenhagen, Denmark; 6 Network Centre for Biomedical Research in Mental Health (CIBERSAM), Health Institute Carlos III (ISCIII), Madrid, Spain; 7 Hospital Univeristari Institut Pere Mata, Reus, Spain; 8 Institut de recerca Biomèdica Catalunya Sud (formerly Institut d’Investigació Sanitària Pere Virgili), Reus, Spain; 9 Universitat Rovira i Virgili, Reus, Spain; 10 FIDMAG Germanes Hospitalàries Research Foundation, Barcelona, Spain; 11 Department of Clinical Medicine, University of Copenhagen, Copenhagen, Denmark

**Keywords:** Diseases, Neuroscience

## Abstract

Childhood maltreatment (CM) has been consistently associated with increased risk and poorer outcomes in bipolar disorder (BD). However, the behavioural and cognitive mechanisms linking CM and suicidality remain poorly understood, limiting the development of targeted preventive interventions. We estimated a regularised partial correlation network to explore the interplay between CM subtypes (emotional, physical, or sexual abuse; emotional and physical neglect), cognitive domains (attention/processing speed, executive function/working memory, socio-emotional cognition, decision-making), impulsivity traits (attentional, motor, non-planning), and suicidal behaviours (ideation, planning, attempts) in 249 euthymic individuals with BD (mean age = 46.14, *SD* = 8.60; 59.84% female; 71.89% BD type I). CM was assessed using the Childhood Trauma Questionnaire (CTQ), cognition with a comprehensive neuropsychological battery, impulsivity with the Barratt Impulsiveness Scale (BIS-11), and suicidality with the Columbia Suicide Severity Rating Scale (C-SSRS) and structured interview. Age, sex, and social desirability (CTQ minimisation/denial subscale) were included as covariates. CM was associated with both cognitive functioning (poorer executive function and working memory, attention/processing speed, and socio-emotional cognition) and suicidality (suicidal ideation and, to a lesser extent, suicide attempts). Emotional abuse was linked to suicidal ideation (strongest association among CM subtypes; *r* = 0.27, *p* <0.001) and showed the highest centrality within the network (strength *z* = 1.76; expected influence *z* = 1.95). All CM subtypes were associated with impulsivity traits. Motor impulsivity emerged as a behavioural bridge between CM and suicidality (bridge expected influence *z* = 0.21), whereas higher socio-emotional cognition, particularly the ability to manage emotions, was associated with fewer suicide attempts. These findings highlight specific cognitive and behavioural mechanisms linking CM and suicidality in BD. Emotional abuse and socio-emotional cognition represent promising targets for trauma-informed and personalised interventions.

## Introduction

Bipolar disorder (BD) is a complex neuropsychiatric condition shaped by genetic and environmental factors [1] and is among the psychiatric disorders most strongly linked to suicide risk, with a lifetime prevalence of suicide attempts of approximately 34%. A meta-analysis involving over 33,000 individuals with BD confirmed this elevated risk, highlighting increased vulnerability among women, those with BD type I, and individuals experiencing rapid cycling [2]. These findings underline the urgent need for improved identification and prevention strategies for suicidal behaviour in BD.

Childhood maltreatment (CM), including abuse and neglect before age 18 [3], represents a major environmental risk factor for the development of psychiatric disorders, including BD [4]. Emotional abuse and neglect, as well as exposure to interparental violence, have shown the strongest associations with the disorder [5]. More than half of individuals with BD report a history of CM [6, 7]. As a form of chronic stress, CM may disrupt neurodevelopmental processes during sensitive periods, leading to structural brain alterations in stress-susceptible regions [8–10] that in turn, increase vulnerability to cognitive, behavioural, and social dysfunction in BD [11, 12]. We previously showed that, in euthymic patients with affective disorders, including BD, higher CM severity was associated with poorer emotion regulation and reduced emotion recognition [13]. Notably, individuals with BD who have a history of suicide attempts report significantly higher levels of CM, both overall and across specific subtypes, compared to non-attempters [14].

More broadly, CM has been robustly associated with suicide behaviour across the lifespan independent of psychiatric condition. Meta-analyses including more than half a million youth and adults indicate that all core CM types significantly increase the risk of suicide attempts (ORs = 1.79 - 3.41 in youth; 2.49 - 3.17 in adults), with sexual abuse showing the strongest effects [15, 16]. CM is also associated with suicidal ideation and planning, with sexual abuse increasing suicide planning risk fourfold and complex abuse showing particularly high risk (OR = 5.18) [15, 16]. A recent meta-analysis estimated that CM accounts for up to 41% of suicide attempts and over 66,000 years of life lost globally [17]. In euthymic individuals with BD, we previously demonstrated that CM constitutes a significant risk factor for suicidal behaviour, and that genetic factors, although associated with CM, did not mediate this relationship [18].

In mood disorders, exposure to CM is associated not only with increased suicidal behaviour but also with cognitive impairments, which in turn appear to exacerbate the risk of *future* suicide attempts [19]. In line with this, a meta‑analysis of 41 studies demonstrated transdiagnostic associations between deficits in impulse control, planning, and working memory and both suicidal ideation and prior suicide attempts [20]. These findings suggest that cognitive impairment may function as an intermediate vulnerability linking early adversity to suicidal behaviour. In BD, however, the literature remains mixed, with past suicide attempts showing positive associations with executive functioning but negative associations with emotion inhibition [20], underscoring the complexity of cognition-suicidality relationships in this population.

A growing body of evidence indicates that CM is robustly associated with cognitive impairment in BD, supporting its role as a potential developmental antecedent of cognitive vulnerability. In our previous work [21] and two recent meta-analyses [11, 12], CM was consistently associated with poorer cognitive functioning in BD, with different CM subtypes exerting distinct cognitive effects. Sexual and physical abuse, as well as physical neglect [21], have been linked to deficits in working memory and executive functions, whereas emotional abuse appears to primarily affect working memory [12]. Such impairments may plausibly increase suicide risk by compromising cognitive control, problem‑solving, and the ability to flexibly regulate distress. However, the relationship between CM and socio‑emotional cognition, defined as the capacity to perceive, understand, and manage emotions, remains unclear [11, 12, 22] even though socio‑emotional dysfunction may be particularly relevant for interpersonal stress reactivity and suicidal behaviour.

Impulsivity represents an additional, closely related pathway through which CM and cognitive dysfunction may increase suicidal risk in BD. Impulsivity is a core feature of BD and a well‑established risk factor for suicide attempts, particularly by weakening the regulation of suicidal ideation and increasing the likelihood of acting on suicidal thoughts [23]. Supporting a developmental link, a meta‑analysis of 55 studies found significant associations between CM and trait impulsivity, with effect sizes ranging from small for sexual abuse to medium‑to‑large for emotional abuse [24]. Moreover, evidence suggests that impulsive aggression may partially mediate the association between CM and suicidal behaviour in BD, accounting for the effects of emotional and sexual abuse [25]. Together, these findings indicate that CM‑related increases in impulsivity, potentially compounded by cognitive deficits, may heighten vulnerability to suicidal behaviour.

Although associations between have been examined, most studies have explored these relationships in *isolation*, typically focusing on specific pairings such as CM and cognition, CM and suicidality, or impulsivity and suicidality, rather than integrating all domains simultaneously. To date, no study has systematically investigated the multidimensional interplay among CM, cognitive impairments, impulsivity, and suicidal behaviour within a unified framework, nor applied network analysis to explore their interconnections in BD and their collective contribution to suicide risk. In the present study, we therefore aimed to estimate direct associations between these variables while controlling for all others using a regularised partial correlation network in a large sample of 249 euthymic individuals with BD. Specifically, we examined whether certain cognitive functions and impulsivity dimensions are more strongly associated with particular forms of suicidal behaviour, thereby providing a more integrated and nuanced understanding of the mechanisms linking CM and suicidality in BD. By modelling these constructs as interconnected components within a network, this approach moves beyond traditional linear analyses and provides a more nuanced understanding of vulnerability pathways. It may also help identify central and bridge nodes linking CM, cognition, impulsivity, and suicidality, thereby informing novel targets for prevention and personalised, precision-based interventions in CM-exposed individuals with BD. Based on previous evidence indicating differential effects of CM subtypes on cognition, social functioning, and suicidal behaviour [11, 12, 15, 16], we hypothesised that: (1) distinct CM subtypes would be differentially associated with deficits in cognitive domains (i.e., working memory, executive functions); (2) cognitive deficits would be linked to particular impulsivity traits; and (3) these impulsivity traits would, in turn, be linked to specific forms of suicidal behaviour.

## Methods

### Participants, ethics, and study design

Participants were recruited from five centres in Barcelona and Tarragona, Spain. At Hospital Pere Mata, Fundació Hospitalària Sant Boi, Fundació Hospitalària Martorell and Fundació Hospitalària Barcelona Nord, recruitment took place between 2016 and 2019 (*n* = 137) [18]. At Hospital Clínic of Barcelona (*n* = 112), recruitment is ongoing as part of two studies on cognition, emotion, and social functioning in CM and BD. Patients who completed assessments for CM, cognitive, impulsivity, and suicidal behaviour were included.

All participants provided written informed consent before study entry. All assessments, including clinical evaluations and CM history, were conducted by trained clinical researchers, psychiatrists, psychologists, and neuropsychologists with prior experience in administering structured clinical interviews and neuropsychological tests. Regular meetings were held to ensure consistency and reliability across study sites. The study adhered the principles of the Declaration of Helsinki and was approved by the Ethics Committee of Hospital Universitari Sant Joan de Reus (15-09-24/9proj1) and Hospital Clínic of Barcelona (HCB/2025/0505; HCB/2026/0049).

Inclusion criteria were as follows: (1) confirmed diagnosis of BD type I or II according to the Diagnostic and Statistical Manual of Mental Disorders (DSM-IV or DSM-5); (2) age between 18 and 60 years; (3) euthymia, defined as a Hamilton Depression Rating Scale (HDRS) [26] score ≤ 8 and a Young Mania Rating Scale (YMRS) [27] score ≤ 6 at the time of assessment and during the 3 months before study inclusion; (4) at least two affective episodes during the course of illness; (5) Caucasian ethnicity; and (6) adequate comprehension of the study procedures and provision of written informed consent.

Exclusion criteria were: (1) intelligence quotient (IQ) < 85 as measured by the Wechsler Adult Intelligence Scale-Third Edition (WAIS-III) [28]; (2) history of neurological disease, brain trauma, or vascular risk factors for cognitive impairment; (3) severe physical illness (e.g., cancer); (4) presence of any other current psychiatric disorder; and (5) being a first-degree relative of another participant.

### Measures

#### Sociodemographic and clinical assessment

All participants were interviewed to obtain sociodemographic and clinical information. To determine eligibility, patients were assessed using the Structured Clinical Interview for the DSM-IV or DSM-5 (SCID-IV or SCID-5) [29, 30]. The HDRS [26] and YMRS [27] were administered to confirm the absence of current depressive or manic episodes.

#### Childhood maltreatment

The presence of CM before the age of 18 was assessed using the Spanish version [31] of the Childhood Trauma Questionnaire–Short Form (CTQ-SF) [32], a 28-item self-report instrument. The CTQ-SF evaluates five CM subtypes: emotional abuse, physical abuse, sexual abuse, emotional neglect, and physical neglect, using a 5-point Likert scale (1 = *never true* to 5 = *very often true*) [33]. Subscale scores were calculated by summing the corresponding five items, yielding a total CM score ranging from 25 to 125, with higher scores indicating greater maltreatment severity. The minimisation/denial subscale [34] was used to detect socially desirable or false-negative response patterns.

#### Cognitive functioning

All participants completed a standardised neuropsychological battery assessing key cognitive domains relevant to BD during euthymia. Neuropsychological assessments were administered by trained researchers, neuropsychologists, following standardised procedures, in a quiet and controlled clinical setting, and according to the administration guidelines of each instrument to ensure consistency across participants. Scores were adjusted for age and education based on normative data, with higher scores indicating better performance across all domains. The battery comprised four cognitive domains:

##### Attention and processing speed

derived from performance on the Continuous Performance Test–II (CPT-II, version 5) [35]; the Trail Making Test Part A (TMT-A) [36] and the Digit Span Forward, Digit Symbol and Symbol Search subtests of the Wechsler Adult Intelligence Scale- Third Edition (WAIS-III) [28].

##### Working memory and executive functioning

derived from performance on the Digit Span Backward, Arithmetic and Letter-Number Sequencing subtests of the WAIS-III [28]; the Stroop Colour-Word Test [37]; the Trail Making Test Part B (TMT-B) [36], and the computerised version of the Wisconsin Card Sorting Test (WCST) [38];

##### Socio-emotional cognition

encompassing processes involved in perceiving, understanding, and regulating emotions, assessed using the Mayer-Salovey-Caruso Emotional Intelligence Test (MSCEIT) [39], which includes four subdomains: perceiving emotions, facilitating thought, understanding emotions, and managing emotions.

##### Decision-making

emotion-based decision-making under uncertainty, reflecting the ability to balance rewards and punishments to maximise long-term gains, was assessed using the Iowa Gambling Task (IGT) [40]. A total decision-making score was calculated [(Deck C + Deck D) − (Deck A + Deck B)].

A detailed methodological framework for the assessment of cognitive domains included in this study is provided in Annex 1 in the Supplementary Materials.

#### Impulsivity

Trait impulsivity was assessed using the 30-item self-report Barratt Impulsiveness Scale (BIS-11), which based on the principal-component structure, comprises three subscales: attentional impulsivity (tolerance for cognitive complexity and persistence), motor impulsivity (tendency to act rashly), and non-planning impulsivity (lack of future orientation). Items are rated on a 4-point Likert scale ranging from 1 (*rarely/never*) to 4 (*almost always/always*), yielding total scores between 30 to 120, with higher scores indicating greater impulsivity [41].

#### Suicidal behaviour

For most participants (*n* = 211), suicidal behaviour was assessed using the Spanish version of the Lifetime/Recent Columbia Suicide Severity Rating Scale (C-SSRS) [42], which evaluates lifetime history of suicidal ideation, planning, and attempts. The severity of suicidal ideation is rated on a 5-point scale: 1 = *wish to be dead*; 2 = *non-specific thoughts*; 3 = *thoughts with method*; 4 = *intent*; 5 = *intent with plan*. Actual, aborted, and interrupted attempts, as well as preparatory behaviours and non-suicidal self-injury, are rated categorically. For the remaining participants (*n* = 38), the number of suicide attempts was assessed through a structured clinical interview (SCID-IV/SCID-5) [29, 30] and data on suicidal ideation and planning were not available.

### Statistical analyses

All statistical analyses were conducted using R (version 4.5.1).

#### Data preprocessing

Preliminary data processing included handling of missing data and computation of composite cognitive scores. For further details on data preprocessing procedures are provided in Annex 1 in the Supplementary Materials.

#### Descriptive analysis

Descriptive statistics were computed to characterise demographic and clinical variables, as well as measures of cognition, impulsivity, and suicidal behaviour.

#### Association analysis

Spearman’s rho correlations were performed to examine bivariate associations between CM subtypes and cognitive domains, impulsivity traits, and suicidal behaviour.

#### Network analysis

Network estimation was conducted using the R packages *bootnet* and *qgraph* [43]. Edges in the network represent regularised partial correlations between two nodes, controlling for all remaining variables. To minimise potential spurious associations, edge weights were estimated using the graphical Least Absolute Shrinkage and Selection Operator (LASSO), which shrinks small partial correlations toward zero, resulting in more stable and sparse networks. The Extended Bayesian Information Criterion (EBIC) hyperparameter *γ* was set to 0.5, as recommended to balance specificity and interpretability with sensitivity [44].

Age, sex, and social desirability (CTQ minimisation/denial subscale) were included as covariates, consistent with previous studies [10, 45]. Age was calculated based on participants’ date of birth and the date of the first evaluation. Sex was coded as 0 = female and 1 = male. A social desirability score was derived from the sum of the responses to the items 10, 16, and 22 of the CTQ minimisation/denial subscale [34]. Covariates were included as nodes in the network but were excluded from visualisation and from subsequent centrality and bridge analyses.

To identify the most influential nodes, we computed centrality indices, specifically strength (sum of absolute edge weights) and expected influence (sum of signed edge weights), using the *bootnet* package. Bridge centrality metrics were calculated using the *networktools* package to identify nodes that acted as connectors between predefined clusters (CM, cognition, impulsivity, and suicidal behaviour).

The accuracy of edge weights was evaluated using 95% non-parametric confidence intervals derived from 2500 bootstrap replications [43]. The stability of edge weights was assessed using the correlation stability (CS) coefficient derived from case-dropping bootstrapping, which reflects the correlation between edge weights obtained from the full data and from subsets [43]. A CS coefficient greater than 0.25 was considered acceptable, and values above 0.50 indicated good stability.

Additionally, to account for the potential effects of illness duration and pharmacological treatment (lithium), we conducted a post hoc network analysis, following the same methodological approach described above.

## Results

### Participant sociodemographic and clinical characteristics

The sample comprised 249 adults with BD, including 149 females and 100 males, with a mean age of 46.1 (*SD* = 8.6). The majority of participants were diagnosed with BD type I (71. 9%). Overall, participants exhibited low levels of depressive (HDRS; *M* = 3.2, *SD* = 3.1) and manic (YMRS; *M* = 1.3, *SD* = 1.7) symptoms at the time of the assessment. The most frequently reported subtypes of CM were emotional neglect (*M* = 10.5, *SD* = 4.9) and emotional abuse (*M* = 9.1, *SD* = 4.8). Further socio-demographic and clinical characteristics of the sample are presented in Table 1. Mean scores and standard deviations *(SD)* for cognitive domains, impulsivity traits, and suicidal behaviours are summarised in Table 2.

**Table 1:** Demographic and clinical characteristics and frequencies of CM in the sample.

	M *(SD)* or *n %*
**Demographic variables**	
Sex	
Female	149 (59.84%)
Male	100 (40.16%)
Age (in years)	46.14 (8.60)
Diagnosis	
Bipolar disorder type I	179 (71.89%)
Bipolar disorder type II	70 (28.11%)
Functioning (FAST), total score	18.17 (12.87)
**Clinical characteristics**	
HDRS	3.23 (3.05)
YMRS	1.26 (1.73)
**Childhood maltreatment**	
CTQ, total score	38.73 (13.78)
Emotional abuse	9.08 (4.75)
Emotional neglect	10.54 (4.89)
Sexual abuse	6.59 (3.53)
Physical abuse	6.30 (2.39)
Physical neglect	6.61 (2.32)
Minimisation/denial	0.64 (0.91)

*CTQ*, Childhood Trauma Questionnaire; *FAST*, Functioning Assessment Short Test; *HDRS*, Hamilton Depression Rating Scale 17-items version; *YMRS*, Young Mania Rating Scale.

**Table 2:** Descriptive statistics of cognition, impulsivity, and suicidality in the sample.

	Mean *(SD)* or *n (%)*
**Cognition**	
*Neurocognition*	
Executive function and working memory, *z*-scores	0.00 (0.69)
Attention and processing speed, *z*-scores	0.00 (0.64)
*Socio-emotional cognition*	
Perceiving emotions	103.40 (20.23)
Facilitating thoughts	104.46 (19.34)
Understanding emotions	99.65 (12.96)
Managing emotions	100.93 (14.24)
Decision-making	14.54 (25.75)
**Impulsivity**	
Cognitive impulsivity	18.79 (3.39)
Motor impulsivity	20.37 (4.52)
Non-planning impulsivity	29.99 (7.26)
**Suicidality**	
Suicide ideation (yes)	2.05 (1.86)
Suicide plans (yes)	24 (9.64%)
Number of suicide attempts	0.42 (0.99)

Sample size is *n* = 249 for all variables except suicide plans and suicide ideation (*n* = 211).

### Associations between CM, cognition, impulsivity, and suicidal behaviour in BD

Bivariate correlations between CM and key study variables are presented in Table 3. Overall, the strongest and most consistent positive correlations were observed between all subtypes of CM and impulsivity traits. Regarding cognitive domains, executive function and working memory, attention and processing speed, and socio-emotional cognition (managing emotions) were negatively associated with several subtypes of CM. CM was also positively associated with suicidal behaviour, particularly emotional abuse and suicide ideation.

**Table 3:** Bivariate correlations between CM subtypes and cognition, impulsivity, and suicidality.

Variables	Emotional abuse	Physical abuse	Sexual abuse	Emotional neglect	Physical neglect
**Cognition**					
*Neurocognition*					
Executive function and working memory	−0.05	−0.13*	−0.14*	−0.13*	−0.14*
Attention and processing speed	−0.06	−0.21***	−0.21**	−0.08	−0.14*
*Socio-emotional cognition*					
Perceiving emotions	−0.03	0.04	0.02	−0.08	−0.09
Facilitating thoughts	0.01	−0.01	0.02	−0.04	0.01
Understanding emotions	−0.01	−0.07	−0.09	−0.10	−0.14*
Managing emotions	−0.11	−0.03	−0.06	−0.20**	−0.22***
Decision-making	−0.01	0.02	0.02	0.01	0.00
**Impulsivity**					
Cognitive impulsivity	0.29***	0.09	0.21***	0.29***	0.16*
Motor impulsivity	0.25***	0.13*	0.25***	0.14*	0.07
Non-planning impulsivity	0.16*	0.20**	0.18**	0.25***	0.16*
**Suicidality**					
Suicide ideation	0.27***	0.07	0.14*	0.18**	0.01
Suicide plans	0.11	0.06	0.14*	0.10	−0.03
Number of suicide attempts	0.15*	0.04	−0.06	0.05	0.02

Sample size is *n* = 249 for all variables except suicide plans and suicide ideation (*n* = 211); *p* < 0.05 (*), *p* < 0.01 (**), *p* < 0.001 (***).

### Network of CM, cognition, impulsivity, and suicidal behaviour in BD

**Fig. 1:**
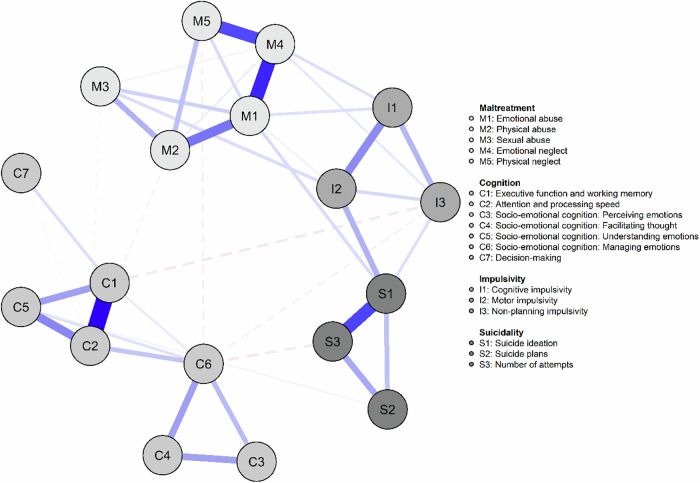
Regularised partial correlation network including overall CM, cognition, impulsivity, and suicidality in BD. Nodes represent variables and edges represent partial correlations. Red and dashed edges indicate negative associations. Sample size is *n* = 249 for all variables except suicide plans and suicide ideation (*n* = 211).

The estimated network included 18 nodes representing CM subtypes, cognitive domains, impulsivity traits, and suicidal behaviour (Fig. 1). The network was relatively sparse, with 43 non-zero edges (28.1% of all possible connections).

Distinct patterns emerged among CM, cognitive functioning, impulsivity, and suicidal behaviour. CM and cognitive nodes were weakly interconnected, with one negative edge between physical abuse and executive function and working memory, and another between physical neglect and socio-emotional cognition (managing emotions). Only one direct connection was observed between CM and suicidal behaviour, linking emotional abuse and suicide ideation. In contrast, multiple connections were found between CM subtypes and impulsivity traits. Neither CM nor impulsivity nodes were directly associated with suicide planning or attempts. Instead, socio-emotional cognition (managing emotions) showed a negative association with the number of suicide attempts.

#### Centrality indices

**Fig. 2:**
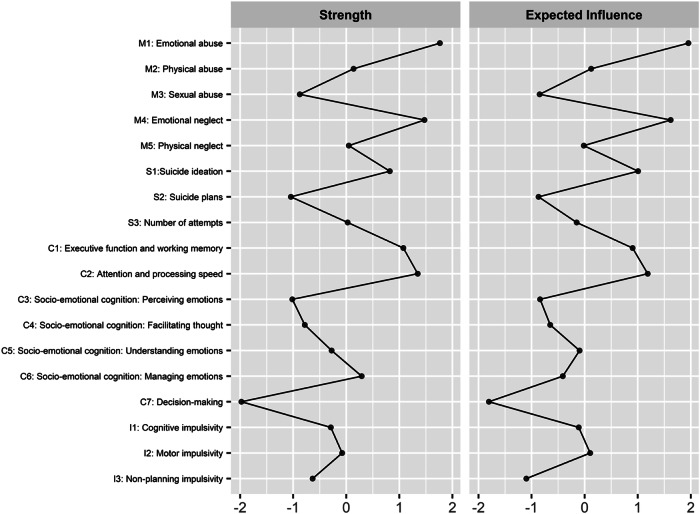
Centrality plots displaying the strength and expected influence for each node in the network. Sample size is *n* = 249 for all variables except suicide plans and suicide ideation (*n* = 211).

**Fig. 3:**
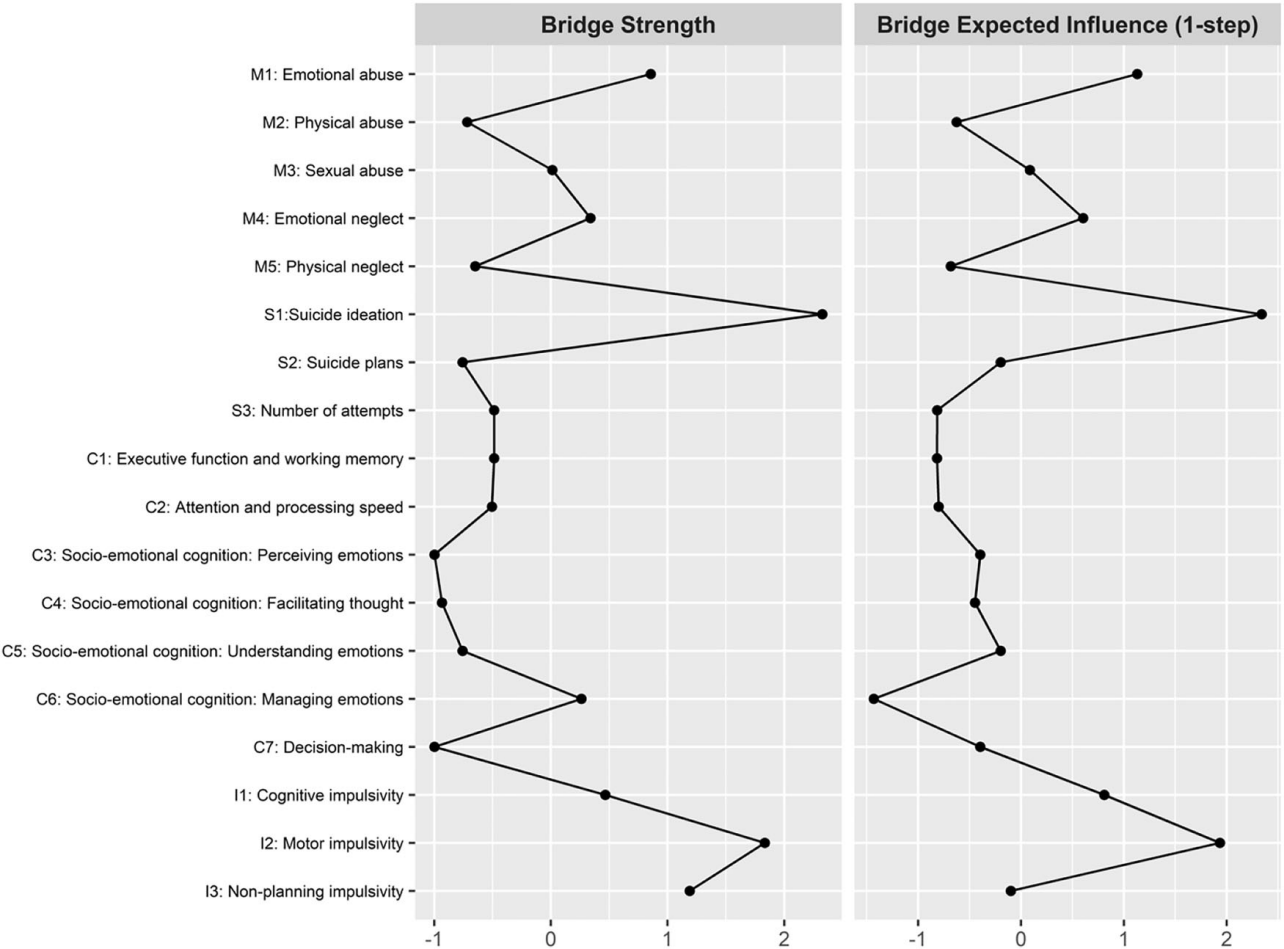
Centrality plots displaying the bridge strength and bridge expected influence for each node in the network. Sample size is *n* = 249 for all variables except suicide plans and suicide ideation (*n* = 211).

Figures 2 and 3 illustrate the results of the centrality analyses (see also Tables S1 and S2, Annex 2, Supplementary Materials). Emotional abuse emerged as the most central node in the network, showing the highest strength (*z* = 1.76) and expected influence (*z* = 1.95) values.

Bridge centrality analyses revealed that impulsivity traits, particularly motor impulsivity (bridge strength; *z* = 0.21, bridge expected influence; *z* = 0.21), acted as key connecting nodes between CM and suicidal behaviour. Additionally, suicidal ideation showed high bridge centrality (bridge strength; *z* = 0.25, bridge expected influence; *z* = 0.25), linking suicidal behaviour with other network domains.

#### Network stability

Edge-weight and centrality estimates demonstrated good stability (CS > 0.50), supporting the robustness of the network structure. Bootstrapped edge-weight and centrality plots are available in the Supplementary Materials (Figures S1 to S4, Annex 3, Supplementary Materials).

#### Additional post hoc network analysis

The inclusion of illness duration and lithium use did not alter the network structure. Some edges were slightly stronger after adjustment for these variables. Overall, the findings remained consistent (Figure S5, Annex 3, Supplementary Materials).

## Discussion

We examined the interplay among CM, cognition, impulsivity, and suicidal behaviour within a unified network framework in euthymic adults with BD. The findings revealed a selective pattern of associations rather than widespread interconnections, suggesting specific behavioural and cognitive mechanisms that may underlie suicidal vulnerability in BD.

Consistent with previous evidence [12], modest negative associations were observed in the bivariate analyses between most forms of abuse and neglect and executive functioning and working memory, while physical and sexual abuse were related to poorer attention and processing speed. Emotional abuse and neglect were linked to lower socio-emotional cognition, particularly in managing emotions. All CM subtypes were positively associated with impulsivity traits, and most (except physical abuse and neglect) were related to suicidal ideation and planning. Notably, only emotional abuse was positively associated with suicide attempts, suggesting its distinctive contribution to suicide risk [46–48], which was further confirmed by its high centrality within the network.

In contrast to the broader associations observed in the bivariate analyses, the network analysis indicated more selective connections between CM and cognitive domains, with limited overlap between specific CM subtypes, particularly physical abuse and neglect, and cognitive performance, consistent with our first hypothesis and previous meta-analytic findings [12]. In line with our second hypothesis, specific cognitive functions were linked to impulsivity traits, especially non-planning impulsivity. Direct connections between CM and suicidal behaviour were scarce, primarily involving emotional abuse and suicidal ideation, consistent with evidence that emotional and sexual abuse exert stronger effects on suicidal risk than physical abuse or neglect [48].

Several connections emerged between CM subtypes and impulsivity traits, supporting the idea that impulsivity may act as a behavioural pathway linking CM experiences with suicidal risk [49]. Among impulsivity dimensions, motor impulsivity appeared particularly relevant, reflecting a tendency to act without deliberation and difficulty inhibiting prepotent responses [50]. These features are closely associated with self-control deficits and maladaptive behaviour. Notably, suicidal ideation showed high bridge centrality, underscoring its role as key connecting node between suicidal behaviour, impulsivity, and cognitive domains, and reinforcing its value as a proximal marker of suicide risk [51].

Unexpectedly, no associations or interconnections were observed between CM and suicidality in emotion-based decision-making under uncertainty. Instead, it appears that socio-cognitive skills, particularly emotion management, play a more prominent role, suggesting that difficulties may relate less to decision-making itself and more to emotion regulation processes. In line with this, poorer socio-emotional cognition was associated with a higher number of suicide attempts, indicating that enhancing emotion-regulation capacity may exert a protective effect against suicidal behaviour. This aligns with evidence indicating that higher emotional intelligence and emotion-regulation skills serve as protective factors against suicidal acts [52].

Centrality analyses further identified emotional abuse as the most influential and interconnected variable within the network, exerting influence across multiple domains. This finding reinforces the central role of emotional abuse as a focal risk factor and supports previous work identifying it as one of the most enduring forms of maltreatment, with broad emotional, behavioural, and social consequences in BD [11, 53, 54].

Although the estimated network was relatively sparse, it demonstrated good stability and reliability, with adequate correlation-stability coefficients (CS > 0.50). This indicates that the observed structure was robust and unlikely to result from sampling variability. While the sample size was moderate, previous simulation studies suggest that networks of similar complexity can be reliably estimated with samples of this magnitude, particularly when stability coefficients exceed recommended thresholds [55].

Overall, this study provides novel evidence of subtle yet consistent behavioural and cognitive mechanisms linking CM to suicidal vulnerability in BD. By identifying emotional abuse as a central focal point of risk, impulsivity, especially its motor component, as a behavioural pathway, and socio-emotional skills as a possible protective factor, our findings contribute to a more nuanced understanding of suicide-risk pathways and support the development of trauma-informed and personalised interventions for individuals with BD.

Taken together, the central role of emotional abuse, motor impulsivity, and socio-emotional cognition highlights a potential pathway linking CM experiences to suicidal vulnerability in BD. Emotional abuse may represent a distal but enduring risk factor that shapes maladaptive emotional and interpersonal patterns, while motor impulsivity may act as a proximal behavioural mechanism facilitating the transition from suicidal thoughts to actions. In contrast, socio-emotional cognitive abilities, particularly emotion regulation, may serve as protective factors by supporting adaptive processing of emotional distress. Considering these domains jointly may help refine risk assessment and inform the development of more targeted, mechanism-based interventions.

### Clinical implications and future research lines

These findings may have relevant clinical implications. Emotional abuse emerged as a central factor associated with suicidal ideation, underscoring the importance of recognising the role of CM in treatment and prevention [56] and of routinely assessing specific CM subtypes in adults with BD. Clinical interventions should address the emotional consequences of such experiences, particularly their links to impulsivity and suicide risk. Although the observed associations were small, the link between socio-emotional cognition and suicide attempts suggests that therapies aimed at improving emotion regulation and interpersonal understanding may help mitigate suicidal vulnerability.

These findings further highlight the potential benefit of interventions in BD that target both the enduring effects of emotional abuse and the mechanisms connecting CM to suicidality. Approaches such as schema or compassion-focused therapy may help reprocess internalised shame and rejection, while training in socio-emotional cognition (e.g., mentalisation-based therapies) can enhance interpersonal functioning. As motor impulsivity appears to bridge maltreatment and suicidality, interventions including dialectical behaviour therapy, mind-body approaches, and mindfulness-based interventions [57] may help strengthen inhibitory control [58] and emotional awareness, supporting adaptive emotional integration.

These insights also point to the need for research and clinical policy to move beyond symptom-based models, adopting integrative frameworks that consider developmental trauma, cognitive-affective functioning, and suicide prevention strategies. Replication through longitudinal designs is required, but the current findings indicate promising directions for future investigation. A key question is whether socio-emotional cognitive skills, particularly those related to emotion management, can be effectively enhanced to reduce suicidal vulnerability in patients exposed to CM.

Given that emotional abuse was directly linked to suicidal ideation, and motor impulsivity served as a bridge between CM and suicidal behaviour, interventions jointly targeting emotion regulation and impulsivity may be particularly effective in enhancing resilience [59] and reducing suicidal risk in BD. Future studies should also examine whether continuous outcome monitoring and treatment prediction can be improved by focusing on impulsivity dimensions most strongly associated with suicidality. These lines of investigation of investigation could clarify the contribution of CM to clinical and functional outcomes and guide the development of more effective, tailored, and trauma-informed interventions for those with BD [60].

### Strengths and limitations

This study has several methodological strengths, including a well-characterised clinical sample of individuals meeting diagnostic criteria for BD and normal IQ. Standardised instruments were used to assess cognition, impulsivity, and suicidal behaviour, along with a structured clinical interview to confirm diagnosis and suicidality. Another notable strength was the control for the CTQ minimisation/denial subscale, which is rarely reported in studies of this kind [34]. Although the associations observed were small, they were consistent with meta-analytic evidence linking CM to cognitive and social outcomes in BD [11, 12]. Importantly, this study went beyond examining pairwise relationships by modelling the interconnections among CM subtypes, cognitive domains, impulsivity traits, and suicidal behaviours within a unified framework. This network-based approach provides richer information on how these variables interact, offering a more integrative perspective that may inform future prevention and intervention strategies.

A key limitation is that the cross-sectional design precludes conclusions about causality. Nevertheless, conditional independence relationships, such as those encoded by partial correlations in network analyses, can offer insight into potential causal structures [44]. Thus, the present findings should be considered exploratory, serving to generate hypotheses for future longitudinal or experimental studies that allow stronger causal inferences. Second, the sample consisted of treatment-seeking individuals with BD recruited from four outpatient units in urban Catalonia, and replication in samples with different demographic or clinical profiles is warranted. Furthermore, the inclusion of euthymic individuals with sustained low levels of depressive and manic symptoms may have resulted in a relatively stable subgroup of individuals with BD, which may limit the generalisability of the findings to more symptomatic or acute populations. Third, the retrospective assessment of CM may be subject to recall bias [61]; however, empirical evidence supports the reliability and validity of retrospective CM reports in BD samples [62]. Fourth, the study did not account for timing or other potentially relevant types of maltreatment, despite their possible impact on socio-cognitive outcomes in BD [5, 63]. Future research should incorporate experiences such as domestic violence and bullying, using standardised definitions and measures of CM [64] that capture the onset, duration, and context of these experiences [65]. Although this study assessed key cognitive domains, impulsivity traits, and suicidal behaviour, it did not include all potentially relevant aspects. Future research should integrate broader assessments of socio-cognitive and real-world functioning [66, 67] which may further clarify the behavioural pathways linking CM, cognition, and suicide risk in BD. Finally, the sample size did not allow for the investigation of network differences between BD subtypes. While post hoc analyses showed that clinical variables including lithium use and illness duration did not considerably change the structure of the network, other relevant clinical variables such as hopelessness that may influence the observed associations were not included in the present analyses. Future studies should incorporate these variables to provide a more comprehensive understanding of the mechanisms underlying suicidality in BD.

## Conclusion

This network analysis of euthymic individuals with BD offers new perspectives on the complex relationships among CM, cognition, impulsivity, and suicidal behaviour. Although the observed associations were small, emotional abuse emerged as the most central and influential subtype of maltreatment, directly associated with suicidal ideation, while motor impulsivity acted as a behavioural bridge linking CM and suicidal behaviour. In contrast, socio-emotional cognition, particularly the ability to manage emotions, was inversely associated with suicide attempts, suggesting a potential protective role for emotion-regulation capacity. These findings underscore the importance of systematically assessing specific maltreatment subtypes and impulsivity dimensions in clinical settings. Interventions that enhance emotion regulation and impulse control, within trauma-informed and personalised care frameworks, may help reduce suicidal vulnerability and improve functional outcomes in individuals with BD. By identifying these interconnections, this study contributes to a more integrated understanding of how early adverse experiences may shape suicide-risk pathways in BD and could inform future approaches to prevention and intervention.

## Supplementary information

Supplementary Materials

## Data Availability

The dataset and R codes supporting the conclusions of this article is available from the first authors. The data are not publicly available due to privacy restrictions.
