# Supplementary material for: Mapping behavioural mechanisms linking childhood maltreatment, cognition, impulsivity, and suicidality in bipolar disorder: A network approach
Source: Transl Psychiatry. 2026 Jun 20;16:317. doi: 10.1038/s41398-026-04177-1 (PMC13283214; doi:10.1038/s41398-026-04177-1)
Supplement: Supplementary file 1 — Supplementary Materials [file 41398_2026_4177_MOESM1_ESM.docx]

**Supplementary Materials**

**Mapping behavioural mechanisms linking childhood maltreatment, cognition,**

**impulsivity, and suicidality in bipolar disorder: A network approach**

[Annex 1. Methods 1](#_Toc227618234)

[Cognitive domains 1](#_Toc227618235)

[Composite score calculation 2](#_Toc227618236)

[Missing data 2](#_Toc227618237)

[Annex 2. Tables 3](#_Toc227618238)

[Annex 3. Figures 5](#_Toc227618239)

# **Annex 1. Methods**

## Cognitive domains

**Neurocognition**


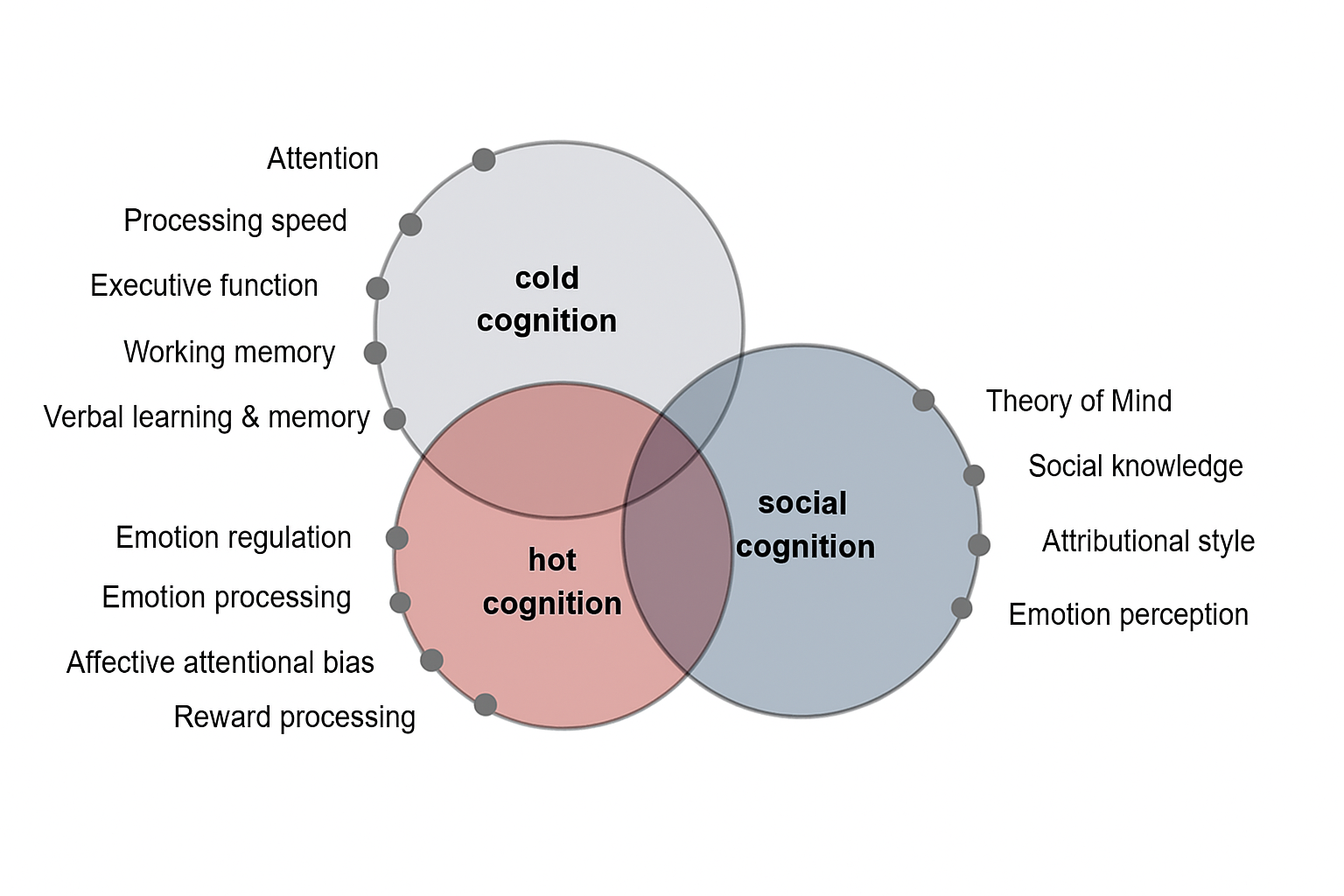


**Socio-emotional cognition**

## Composite score calculation

Two cognitive composite scores were computed: *Executive function and working memory* and *attention and processing speed* using the performance scores on the neurocognitive tests listed in section 2.2. Prior to creating these composite scores, reaction time scores from the Trail Making Test Part A and B were inverted such that higher scores consistently reflected better performance. Cognitive scores were standardised (z-scores), and to limit the influence of extreme observations, values were winsorised to fall within the range of –4 to +4 (Osborne, 2010).

## Missing data

Missing data ranged from 3.3% to 20.9% across variables and were handled via multiple imputation with chained equations (*m* = 60) with the R package *mice* (van Buuren & Groothuis-Oudshoorn, 2011). The number of imputations was set to approximate the proportion of participants having any missing data, in line with recommended practice (White et al., 2011). Missing-at-random tests indicated that missingness was not systematically associated with clinical characteristics.

# **Annex 2. Tables**

| **Table S1.** *Standardised centrality z-scores* | | |
| --- | --- | --- |
|  | Strength | Expected influence |
| Emotional abuse | 1.76 | 1.95 |
| Physical abuse | 0.14 | 0.12 |
| Sexual abuse | -0.88 | -0.85 |
| Emotional neglect | 1.47 | 1.62 |
| Physical neglect | 0.05 | -0.02 |
| Suicide ideation | 0.82 | 1.01 |
| Suicide plans | -1.04 | -0.87 |
| Number of attempts | 0.03 | -0.15 |
| Executive function and working memory | 1.08 | 0.90 |
| Attention and processing speed | 1.25 | 1.19 |
| Perceiving emotions | -1.02 | -0.84 |
| Facilitating thought | -0.78 | -0.65 |
| Understanding emotions | -0.28 | -0.10 |
| Managing emotions | 0.29 | -0.41 |
| Decision-making | -1.98 | -1.81 |
| Cognitive impulsivity | -0.29 | -0.11 |
| Motor impulsivity | -0.08 | 0.10 |
| Non-planning impulsivity | -0.63 | -1.10 |
|  |  |  |

| **Table S2.** *Standardised bridge centrality z-scores* | | |
| --- | --- | --- |
|  | Strength | Expected influence |
| Emotional abuse | 0.14 | 0.14 |
| Physical abuse | 0.02 | -0.02 |
| Sexual abuse | 0.08 | 0.04 |
| Emotional neglect | 0.10 | 0.09 |
| Physical neglect | 0.03 | -0.03 |
| Suicide ideation | 0.25 | 0.25 |
| Suicide plans | 0.02 | 0.02 |
| Number of attempts | 0.04 | -0.04 |
| Executive function and working memory | 0.04 | -0.04 |
| Attention and processing speed | 0.04 | -0.04 |
| Perceiving emotions | 0.00 | 0.00 |
| Facilitating thought | 0.00 | 0.00 |
| Understanding emotions | 0.02 | 0.02 |
| Managing emotions | 0.09 | -0.09 |
| Decision-making | 0.00 | 0.00 |
| Cognitive impulsivity | 0.11 | 0.11 |
| Motor impulsivity | 0.21 | 0.21 |
| Non-planning impulsivity | 0.16 | 0.03 |
|  |  |  |

# **Annex 3. Figures**

| ***Figure S1.*** Bootstrapped confidence intervals of estimated edge-weights |
| --- |
| 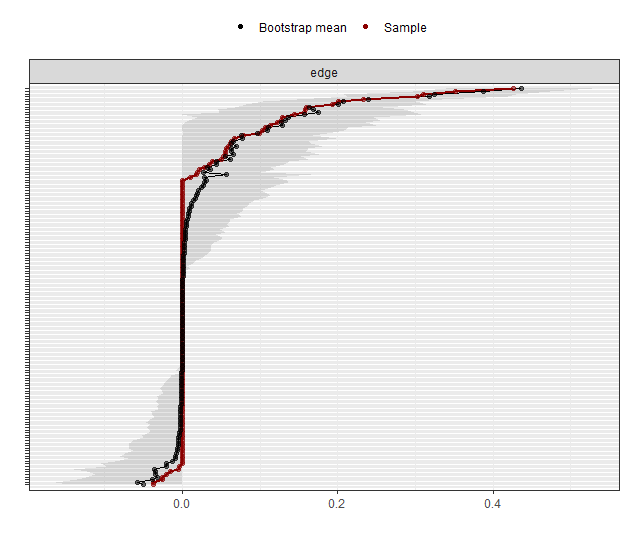 |
| *Note.* The horizontal lines represent edges, arranged from the strongest edge weight at the top to the weakest at the bottom. Red lines show the sample values, black lines represent the bootstrapped values, and the grey shaded area illustrates the 95% bootstrapped confidence intervals. |

| ***Figure S2.*** Bootstrapped edge weight difference tests |
| --- |
| 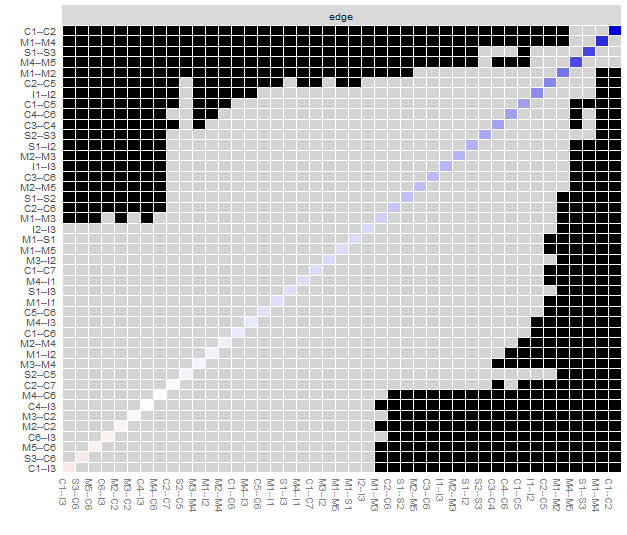 |
| *Note.* Black squares indicate pairs of edges that differ significantly. An alpha level of 0.05 was used. |

| ***Figure S3.*** Stability of strength centrality for the network |
| --- |
| 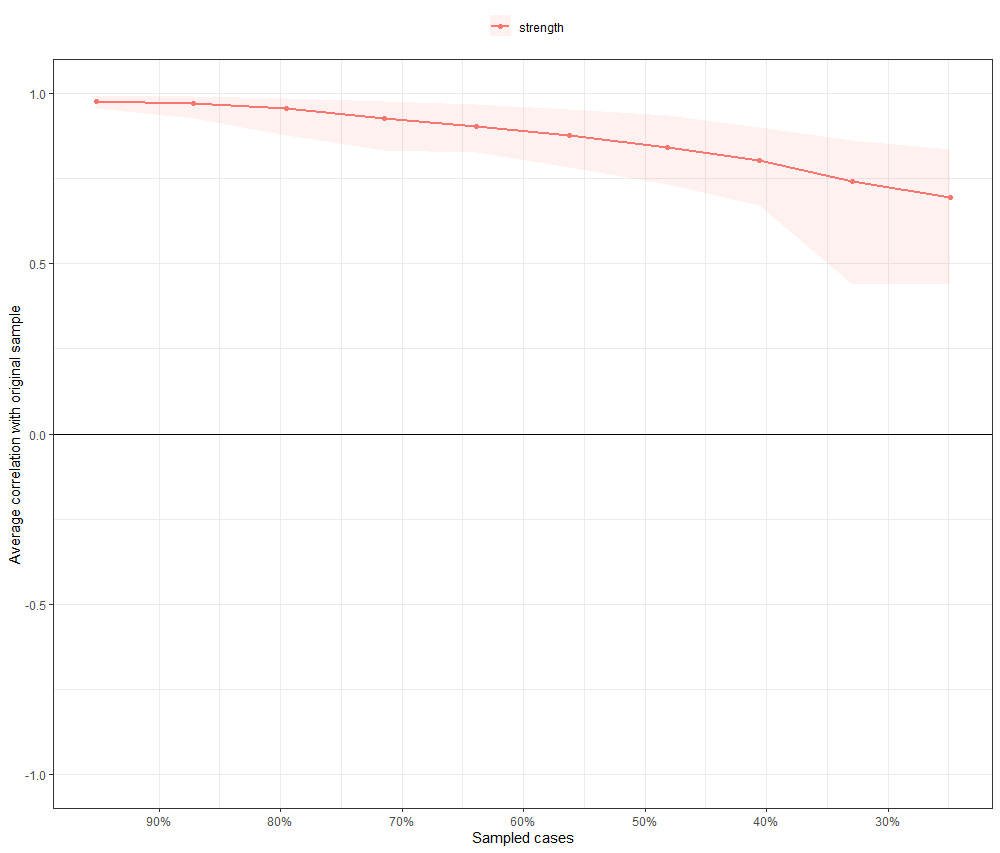 |
| *Note.* Stability is assessed by calculating the average correlation between strength centrality in the original network and in networks generated after removing cases. The solid line represents the mean correlation, and the shaded area shows the 2.5th–97.5th quantile range. |

| ***Figure S4.*** Stability of edge centrality for the network |
| --- |
| 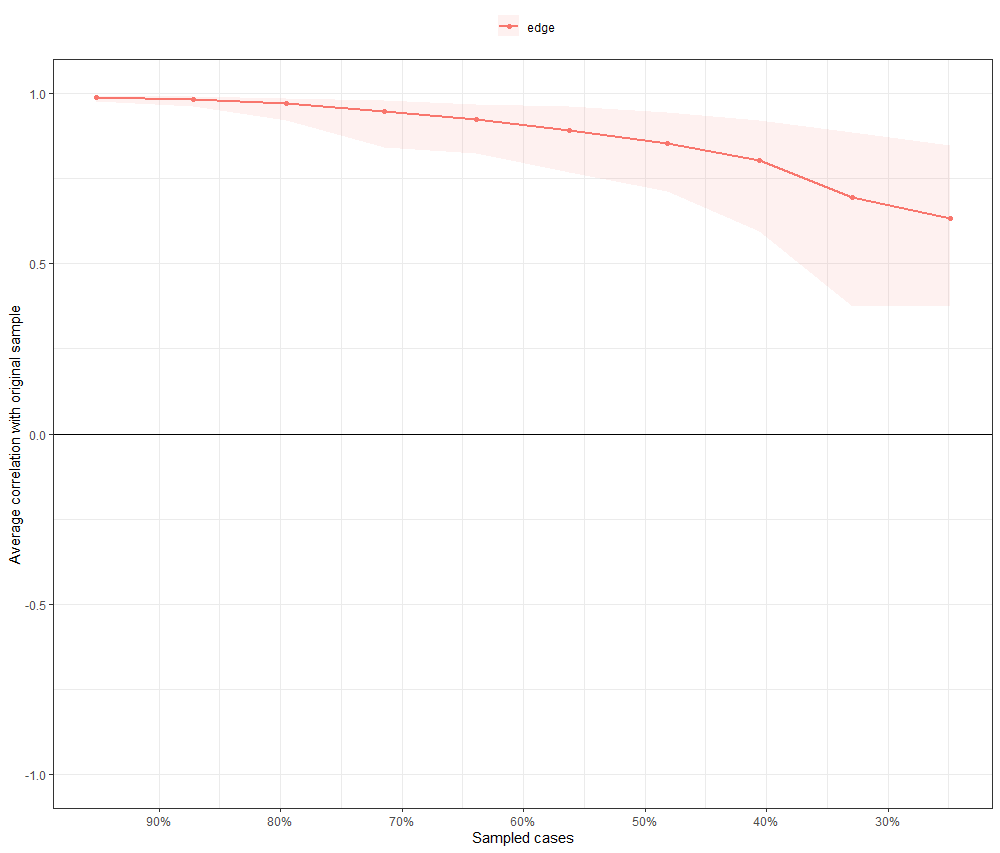 |
| *Note.* Stability is assessed by calculating the average correlation between the edge weight order in the original network and in networks generated after removing cases. The solid line represents the mean correlation, and the shaded area shows the 2.5th–97.5th quantile range. |

***Figure S5.*** Regularised partial correlation network adjusted for lithium use and illness duration


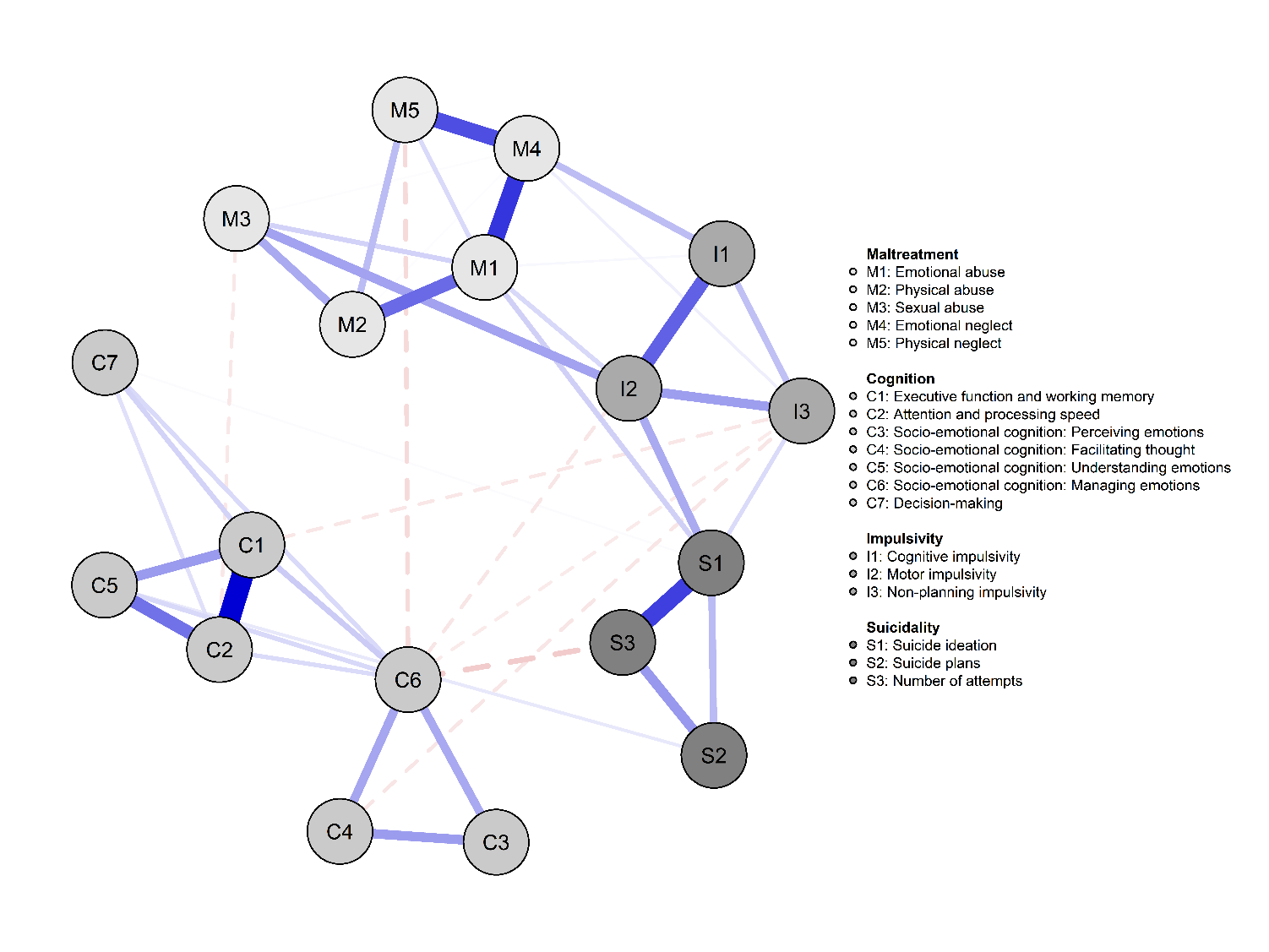


*Note.* Nodes represent variables and edges represent partial correlations. Red and dashed edges indicate negative associations. Sample size is *n* = 249 for all variables except suicide plans and suicide ideation (*n* = 211).

**References**

Osborne, J. W. (2010). Data Cleaning Basics: Best Practices in Dealing with Extreme Scores. *Newborn and Infant Nursing Reviews*, *10*(1), 37–43. https://doi.org/10.1053/j.nainr.2009.12.009

van Buuren, S. van, & Groothuis-Oudshoorn, K. (2011). mice: Multivariate Imputation by Chained Equations in R. *Journal of Statistical Software*, *45*, 1–67. https://doi.org/10.18637/jss.v045.i03

White, I. R., Royston, P., & Wood, A. M. (2011). Multiple imputation using chained equations: Issues and guidance for practice. *Statistics in Medicine*, *30*(4), 377–399. https://doi.org/10.1002/sim.4067
